# Report of the Malignant Fever, Called the Pali Plague

**Published:** 1840-01

**Authors:** 


### Art. XII.

*Report of the Malignant Fever, called the Pali Plague, which has prevailed in some parts of Rajpootana, since the month of July,* 1836. *Prepared and published by order of the Government of India, under the direction of the Bengal Medical Board.* By James Ranken, m.d., Officiating Secretary to the Board.—*Calcutta*, 1838. 8vo, pp. 231.

We consider this an interesting Report of a destructive fever which first appeared in the principality of Joudpore or Marwar, at Pali, a considerable town, reckoned the emporium of the trade between Central India and the seaport of Guzerat. This fever was regarded as a "nova pestis" in the region in which it appeared, and, by the majority of observers, it was considered identical with the plague of the Levant. From this latter opinion, Dr. Ranken departs, and, as it appears to us, on sufficient grounds.

The following is Dr. Ranken's picture of the epidemic, drawn, as he expresses it, from the descriptions of all the medical observers :

"No sense of indisposition gives warning of the approach of this disease. It comes on with slight rigour, headache, nausea, and pain in the loins. The skin soon grows hot and dry, the pulse from 130 to 150 in the minute, is soft and easily compressible. The tongue is variously covered with white, light brown, or darkish fur, and these colours are sometimes intermixed. Vomiting and irritability of stomach occur, though rarely. The bowels are bound; the abdomen tumid and full, is seldom painful on pressure. The eyes appear heavy, maudlin, and bloodshot, and occasionally look as if injected with lake. The countenance is expressive of anxiety and inward pain, the respiration apparently unaffected by the predominant malady, is often impeded by concomitant inflammation of the lungs. Glandular swellings appear in the groins, armpits, and neck, most frequently on the left side, as also under the jaws and ears, and in the upper part of the thigh. They are generally perceptible on the first or second day, and rarely increase above the size of a walnut, but in some instances they become much larger, burst, and discharge purulent matter. The symptoms are sometimes all so mild, that the sick keep walking about till they gradually recover. In most of the cases observed, there was a visible abatement of the disease, every twenty-four hours, towards morning; but in the worst forms of it, intense fever continued night and day, without any remission; the patients could not rise from their cots, on account of extreme debility, and an attempt to raise them to the erect posture produced fainting. Hemorrhage from the lungs took place in a few, and was much dreaded. Delirium occurred rarely at the beginning, but coma generally supervened shortly before death. When violent, the malady ran its fatal course in three days; when mild, with or without the affection of the glands, it was protracted to fifteen or twenty days, like the ordinary fevers of the country." (pp. 15, 16.)

These symptoms (independent of discrepancies of no essential moment) constituted, we are told, in every place visited by medical officers, the Pali disease. Dr. Ranken proceeds to examine whether they suffice to determine that the disease was the real plague; and concludes in the negative, for the following reasons:

1st That most of the external appearances, which Russell and other original observers account pathognomonic of the plague, carbuncles, wheals (vibices), spots, and lividness of parts of the body after death, seem never to have been met with in this disease; and

2dly. That buboes, and other glandular tumours, so far from being certainly diagnostic of the plague, do not appear at all in many marked cases of it, and these the most fatal; and that, moreover, such glandular affections were observed by Drs. Cheyne and Barker in the typhus, which destroyed so many of the lower orders of Irish between 1816 and 1819; by Dr. Rush, in the bilious yellow fever of Philadelphia in 1793; and, as Dr. Ranken informs us, are common in the destructive fever which has arisen occasionally in the remoter parts of the mountainous district of Kumaon for these last fifteen years, and which was particularly fatal in 1834 and 1835.

Any evidence for the foreign origin of the disease, and for its importation into Hindostan, derived from its correspondence in distinctive character with the plague of the Levant, being thus disposed of, the following very refined piece of speculation, to which the annals of the controversy on contagion could furnish many a parallel, receives all the investigation it merits:

"Zerawar Mull, the great loan contractor, banker, and merchant of Rajpootana, having gone to Guzerat, it was alleged, in prosecution of his multifarious business, returned with merchandise which had come from Bhaonuggur or Surat, and stopped several days at Pali, just before the appearance of the fever. Hence the inference confidently drawn, that as those seaports receive goods from the Persian Gulf, the Red Sea, and from Egypt itself, the home of pestilence, Zerawar Mull must have brought the plague to Pali. This wealthy personage, however, it turns out, was religiously and not commercially engaged in a pilgrimage to the temple at Teeruth. He neither bought or sold throughout his journey, and came back perfectly well. Besides, the importation of any articles whatever, from pestilential countries, has not been ascertained in this case: no unusual sickness existed at Bhaonuggur, Surat, or in the provinces connected with them; and the foreign merchandise, purchased in their markets for many parts of western, northern, and central India, was not even suspected of communicating disease anywhere, except at Pali. But numerous small traders, we were again told, availing themselves of Zerawar Mull's escort, came in his train to the emporium of Marwar, and though in perfect health themselves, they might confidingly have purchased unopened bales of Egyptian cloth at Bhaonuggur, and disposed of them to equally unsuspecting retail merchants at Pali, who being the first to unpack the tainted bales, would be the first to suffer from the foreign contagion. There is reason to believe that the only cloths received at the period in question were English cottons; but, at all events, the original purchasers escaped at the opening of the packages, and the printers, who must have subsequently got the pieces to ornament, were the earliest victims of the fever. I need not notice more allegations of the same kind, which nobody would now advance. So long a chain of improbabilities has seldom been devised to support an untenable hypothesis on any subject." (pp. 29, 30.)

Our reading has shown us, that "chains of improbabilities," such as this, are not so rare in controversies on the etiological branch of our art, as Dr. Ranken seems to imagine; but we trust that the diffusion of knowledge, by works like his own, will render them more rare than heretofore, and finally extinguish them.

Dissatisfied, as well he might be, with such an hypothesis on the origin of the disease as the foregoing, the author seeks, and has no difficulty in discovering, endemial causes, capable, under certain atmospheric conditions, of engendering pestilence in abundance. The following passage we consider to be written in the true spirit of medical philosophy:

"The people of the north-western provinces of British India, and of Marwar more especially, are to be considered in varying stages of transition from anarchy and want to comparative plenty and order. They have advanced within reach of many improvements in the physical condition of society; but having made no corresponding acquisitions in intelligence, their minds retain the impress of former barbarity and misrule, and are yet unable to turn the advantages of a better system to account. Hence, animal instinct predominating over foresight and enterprize, population has augmented faster than increasing agriculture and commerce have extended. The hut, or one of the same dimensions, which was the unwholesome den of four persons, for example, fifty years ago, now shelters six human beings, within a space barely sufficient for the accommodation of one. These miserable habitations are often inclosed by an outer screen or wall, for the double purpose of confining the cattle and secluding females from the public gaze. Glass to let in light, or apertures to admit fresh air being unknown, the door, in order to keep out heat at one season and cold at another, is generally shut. The poor feel most at home in such dark places with their children lying round them on the floor, too much like hogs in a sty amidst their litter. The sense of insecurity for life and property, which deterred their ancestors from living near the fields which they cultivated, in farm-houses and cottages scattered over the face of the country, owing to hereditary habits of thinking, still perceptible to this day under the very cannon of Fort William, makes the peasantry everywhere accumulate their hovels within the narrowest limits, and the consequence is, that each dirty village, unventilated, and overcrowded with man and beast, exhibiting every sort of nastiness, is a focus of disease.

"The most obvious means of raising food for the augmenting numbers of inhabitants, involve the cutting down of jungle, the breaking up of waste lands, and the irrigation of new fields for grain, all of which are known by lamentable experience to occasion noxious exhalations. These operations, the necessary attendants on the establishment of a new station, which is a sort of European colony in our provinces, added perhaps to the injudicious choice of a locality, almost in every instance render it unhealthy at first." (pp. 35, 36.)

The reports of the medical officers, who visited the different parts of the infected district, all confirm the general abstract of its condition given by Dr. Ranken.

The author ascribes to malaria the origin of the disease, but admits that human intercourse, or, in other words, contagion, was instrumental in its diffusion. This contagious influence he seems to view as rather contingent on circumstances, such as crowding, imperfect ventilation, and filth, than as abstractedly potential; and hence he remarks that the epidemic depopulated the cottage, but spared the palace. These circumstances, however, existed in abundance in the wretched Indian villages, the original over-crowding being aggravated by the fugitives from Pali, regarding whom it is remarked, that wherever they took up their residence, the fever shortly afterwards appeared. It might be reasonably questioned, whether this influx of strangers, though from an infected district, did not tend to diffuse the disease rather by multiplying sources of malaria than by any direct transmission from individual to individual. The efficacy of certain sanatory cordons, in preventing the diffusion of the disease, furnishes stronger evidence of contagion than any circumstance connected with it.

We consider Dr. Ranken's report a very excellent one, and as bearing a very important and confirmatory relation to Dr. Bowring's paper on plague and quarantine, noticed in our Fifteenth Number.

